# Sero-conversion rate of Syphilis and HIV among pregnant women attending antenatal clinic in Tanzania: a need for re-screening at delivery

**DOI:** 10.1186/s12884-015-0434-2

**Published:** 2015-01-22

**Authors:** John DT Lawi, Mariam M Mirambo, Moke Magoma, Martha F Mushi, Hyasinta M Jaka, Balthazary Gumodoka, Stephen E Mshana

**Affiliations:** Ministry of health and social welfare, Department of Curative services, P.O. Box 9083, Dar esSalaam, Tanzania; Department of Microbiology and Immunology, Weill Bugando School of Medicine, P.O. Box 1464, Mwanza, Tanzania; Evidence for Action Project, P.O. Box 13731, Dar es salaam, Tanzania; Department of Internal medicine, Weill Bugando School of Medicine, P.O. Box 1464, Mwanza, Tanzania; Department of Obstetrics & Gynecology Weill Bugando School of Medicine, P.O. Box 1464, Mwanza, Tanzania

**Keywords:** Syphilis, *Treponema pallidum*, Human immunodeficiency virus, Seroprevalence, Seroconversion, Sexually transmitted infection

## Abstract

**Background:**

Despite the available cost effective antenatal testing and treatment, syphilis and human immunodeficiency virus (HIV) are still among common infections affecting pregnant women especially in developing countries. In Tanzania, pregnant women are tested only once for syphilis and HIV during antenatal clinic (ANC) visits. Therefore, there are missed opportunities for syphilis and HIV screening among those who were not tested during ANC visits and those acquiring infections during the course of pregnancy. This study was designed to determine the syphilis and HIV seroprevalence at delivery and seroconversion rate among pregnant women delivering at Bugando Medical Centre (BMC).

**Methods:**

A cross sectional, hospital-based study involving pregnant women attending Bugando Medical Centre (BMC) antenatal clinic was done from January to March 2012. Serum samples were collected and tested for HIV and syphilis using HIV and syphilis rapid tests. Demographic and clinical data were collected using a standardized data collection tool and analysed using STATA version 11.

**Results:**

A total of 331 and 408 women were screened for syphilis and HIV during antenatal respectively. Of 331 women who screened negative for syphilis at ANC, nine (2.7%) were seropositive at delivery while of 391who tested negative for HIV during ANC eight (2%) were found to be positive at delivery. Six (1.8%) and 23 (9%) of women who did not screen for syphilis and HIV at ANC were seropositive for syphilis and HIV at delivery respectively. There was significant difference of seroprevalence for HIV, among women who tested negative at ANC and those who did not test at ANC (2% vs.9%, P,<0.001). The overall prevalence of syphilis and HIV at delivery was 15 (2.3%) and 48 (7.2%) respectively. Syphilis seropositivity at delivery was significantly associated with HIV co-infection (p < 0.001), male partner circumcision (p = 0.011) and alcohol use among women (p < 0.001).

**Conclusions:**

The current protocol of screening for syphilis and HIV only once during pregnancy as practiced in Tanzania may miss women who get re-infected and seroconvert during pregnancy. Re-screening for syphilis and HIV during the course of pregnancy and at delivery is recommended in Tanzania as it can help to identify such women and institute appropriate treatment.

## Background

Sexually transmitted infections (STIs) are of public health concern in many developing countries including Tanzania. Syphilis and HIV have been of a major concern because of their impact on pregnancy outcome [1]. Syphilis is caused by the bacteria *Treponema pallidum,* and is categorized as one of the genital ulcerative disease [2,3] while HIV/AIDS is caused by human immunodeficiency virus(HIV). Pregnant women are at high risk of syphilis infection due to pregnancy induced cervical changes, such as hyperaemia, eversion and friability which facilitate easy transmission of syphilis leading to spirochaetaemia [4].

The World Health Organization (WHO) estimates that about 2 million pregnant women are infected by *T.pallidum* globally with a 45-70% probability (1.2 million cases) of vertical transmission to neonates annually [5,6]. In developing countries, 3-15% of women of child-bearing age are infected with syphilis [3,7]. Syphilis screening during pregnancy is important strategy to prevent early fetal and neonatal death which is estimated at 620,000 worldwide annually [5].

As syphilis, HIV infection in pregnancy has become a major concern in many developing countries. Previous reports showed that approximately 1.5 million HIV-positive women become pregnant each year [8] with mother to child transmission rate estimated at 600 000 annually [9]. Previous studies showed that the incidence of HIV is higher in pregnant women than in non pregnant women [10]. During pregnancy the risk of HIV acquisition increases due to immunological and hormonal changes, resulting in increasing progesterone levels which affects the mucosa of the genital tract [11,12]. HIV infection during pregnancy has been associated with adverse fetal and maternal outcomes [13].

Early detection and treatment of syphilis and HIV in pregnancy, especially when done before the second trimester, is essential in preventing long term neonatal complications and transmission of disease [3]. Even pregnant women infected with *T.pallidum* four weeks before delivery can still result in birth of an affected newborn [4]. Moreover, HIV seronegative women who seroconvert in late pregnancy can have maternal fetal transmission [14]. It is recommended that syphilis screening done at the first antenatal clinic (ANC) visit in the first trimester of pregnancy, third trimester for detection of new infections acquired during pregnancy and at delivery for those who missed the opportunity of antenatal screening to allow for an early diagnosis and treatment of infections in women and newborns [2]. Similarly HIV testing in early and late pregnancy or at delivery reduces the risk of mother to child transmission [15].

However, in developing countries, syphilis testing during antenatal is low, ranging from 30 to 38%. As a result, few women get tested and treated for infections acquired during pregnancy [3]. In Tanzania, women are routinely screened for syphilis and HIV once in the entire pregnancy. The ANC attendance in Tanzania is more than 95% with about 60% hospital delivery. About 42% of women attend ANC at least four times and only 15% of women starts ANC before sixteen weeks of gestation [16]. Thus many women are likely to progress up to delivery without being screened for syphilis and HIV. It is important to establish seroprevalence of these infections among pregnant women at initiation of pre natal care and at delivery in order to evaluate if the single screening method at initiation of prenatal care is effective in detection and control of these infections. In addition, in developing countries including Tanzania, few pregnant women get tested for syphilis during ANC visits, [17] thus the need for screening at delivery of the non reactive women and those who miss the opportunity of screening during pregnancy. Such data is scarce in Tanzania and this study aimed to provide this information to help health policy makers plan for effective strategies to control syphilis and HIV infections during pregnancy.

## Methods

This was cross-sectional hospital based study involving women attending Bugando Medical Centre (BMC) labour ward between January and March 2012. BMC is a consultant and teaching hospital for the Lake and Western zones of the United Republic of Tanzania. It is situated along the shores of Lake Victoria in Mwanza City with a bed capacity of 900. It is a referral centre for specialized tertiary care for eight regions: Mwanza, Geita, Mara, Simiyu, Kagera, Shinyanga, Tabora and Kigoma. It serves catchments population of approximately 13 million people (http://www.bugandomedicalcentre.go.tz).

The study included pregnant women who were not tested or tested negative for syphilis and HIV during antenatal care visits who delivered at BMC during the study period. Sample size for this study was calculated by using a formula of Kelsey et al. 1996 [18]. Proportion of exposed with disease (p_1_) was 3.0% [19] and proportion of unexposed with disease (p2) was = 8.0% [20]. Using the above proportions, power, confidence interval and ratio, the sample size was computed by Open Epi- sample size calculation for cross sectional study to be 327 in each group, making a minimum total sample size of 654. However, in this study 678 pregnant women were recruited.

All eligible consented pregnant women with antenatal card (document showing number of visits and services provided) on admission were recruited into the study until a desired sample size was reached. Women without antenatal card at the time of admission were excluded from the study.

### Ethical consideration

Ethical clearance was sought from the Department of Obstetrics and Gynecology and CUHAS/BMC Research Committee (CREC). A written informed consent was obtained from each participant after explaining the aims of the study and confidentiality was ensured throughout. Syphilis and HIV seropositive women were advised to attend at STI clinics with their partners for treatment, and were also linked to pediatricians for investigations and treatment of their babies. HIV positive women were linked to prevention of mother to child transmission (PMTCT) services for management.

### Data, sample collection and laboratory diagnosis

A questionnaire was used to collect important parameters such as socio demographic data, gynecological and obstetric history and antenatal HIV status of study participants. The information given by patients was verified by crosschecking with antenatal cards information and results for syphilis tests and HIV tests were confirmed from the laboratory database and PMTCT unit. Venous blood (4mls) was taken using plain vacutainer tubes (BD, Nairobi, Kenya) and sent to the laboratory for rapid plasma reagin (RPR) test and reactive samples were confirmed by Immunochromatographic (rapid) treponemal test. Pre-test counseling and testing for HIV were done for pregnant women who had never had HIV testing and those screened negative at ANC visits following TANZANIA NATIONAL GUIDELINES ALGORITHMS.

### Data analysis

The collected data were sorted, entered into Microsoft EXCEL software and then imported into STATA statistical package version 11 (Stata Corp LP College Station, Texas, USA) for consistent checks and cleaning. Analysis was done using the same package. Results were presented in percentages and odds ratio at 95% confidence level. Logistic regression analysis was done to test for the association.

## Results

### Social demographic characteristics of study participants

During the study period, a total of 1599 pregnant women were admitted in the labour ward. Out of 1599 pregnant women, 678 (42.4%) were recruited into the study. The results for 663 women were analyzed. Data for fifteen recruited participants were not analyzed because their laboratory results for syphilis were not found due to misplacement of samples. The median age of participants was 25 (range of 15 – 44 years). A total of 78.4% resided in the urban area while 94.6% of participants had at least primary education (Table 1). Majority of the participants (71.5%) attended ANC at the primary health care facilities (dispensaries and health centre) with 61.5% of the participants made four or more ANC visits (Table 2). A total of 43.2% of women tested for syphilis within 16 weeks of pregnancy. The main reason for others not to test was lack of reagents (46.4%); other reasons include not informed to test and lack of trained personnel to perform a test. No statistical significant difference was obtained among those tested and those not tested when compared to number of visits and whether they are from urban or rural

**Table 1:** **Social demographic characteristics of study participants**

**Characteristics**	**Number = 663 (%)**
**Age-Median [Range]**	25 [15–44]
**Place of residence**	
Rural	143 (21.6)
Urban	520 (78.4)
**Level of education**	
No formal education	36 (5.4)
Primary education	404 (60.9)
Secondary education	196 (29.6)
Higher education	27 (4.1)
**Marital status**	
Single	104 (15.7)
Married	559 (84.3)

**Table 2:** **Antenatal care profile of study participants**

**Characteristics**	**Number (%)**
ANC services facility in index pregnancy, n = 663	
**Dispensary or health centre**	474 (71.5)
**Hospital**	189 (28.5)
GA(weeks) at first ANC visit, n = 663	
**≤16**	297 (44.8)
**>16**	366 (55.2)
ANC visits in index pregnancy, n = 663	
**<4 visits**	255 (38.5)
**≥4visits**	408 (61.5)
GA(weeks) at ANC syphilis screening, n = 331	
**≤16**	143 (43.2)
**>16**	188 (56.8)
Reasons for not testing for syphilis at ANC, n = 332	
**Not informed to do a test**	61 (18.4)
**No reagents**	154 (46.4)
**No personnel to do a test**	117 (35.2)

Of 663 women enrolled, 331 (49.9%) were screened for syphilis during antenatal visit and 408 (61.5%) were screened for HIV during ANC. Of 663 women, 321 and 373 tested negative for syphilis and HIV during antenatal visits respectively. Of 321 who tested negative for syphilis at ANC 9 (2.7%) were seropositive at delivery while for 373 women who screened negative for HIV at ANC 8/373 (2%) were positive at delivery. Among those who did not test for syphilis and HIV at ANC 6 (1.8%) and 15/225 (6.6%) were positive for syphilis and HIV at delivery respectively.

The overall seroprevalence of syphilis and HIV at delivery among study participants were 2.3% and 7.2% respectively (Table 3). Syphilis seropositivity among pregnant women was significantly associated with HIV infection (p < 0.001),uncircumcised male partner (p = 0.011) and woman’s alcohol intake(p < 0.001) (Table 4) while only syphilis positivity was found significantly associated with HIV infection (Table 5). In this study alcohol intake was defined as taking more than three bottles per week.

**Table 3:** **Syphilis and HIV seroprevalence at delivery among study participants**

**ANC syphilis tests**	**Syphilis test results**		**HIV test results**	
	**Positive (%)**	**Negative (%)**	**Positive (%)**	**Negative (%)**
Not tested, n = 332	6 (1. 8)	326 (9.2)		
Tested negative, n = 321	9 (2.8)	322 (97.3)		
			**Positive (%)**	**Negative (%)**
Not tested (255)			15 (6.9)	240 (93.1)
Tested negative (373)			8 (7.5)	365 (92.5)

**Table 4:** **Factors associated with syphilis seropositivity at delivery: Logistic regression analysis**

**Patient’s characteristic**	**Syphilis results**		**Univariate**		**Multivariate**	
	**Positive n (%)**	**Negativen (%)**	**OR [95% CI]**	**p-value**	**OR [95% CI]**	**p-value**
***Age***						
<20 (81)	1 (1.23)	80 (98.7)	1			
≥20 (582)	14 (2.4)	568 (97.6)	1.97 [0.255 - 15.198]	0.515	1.53 [0.179-13.147]	0.69
***Education***						
Informal (36)	2 (5.7)	34 (94.3)	1			
Formal	13 (2.1)	614 (97.9)	2.77 [0.602 - 12.807]	0.190	2.09 [0.410-10.691]	0.37
***HIV status***						
Negative (615)	9 (1.5)	606 (98.5)	1			
Positive (48)	6 (12.5)	42 (87.5)	9.61 [3.28 - 28.35]	0.0001	9.13 [2.889-28.867]	0.0001
***Partner circumcision***						
Circumcised (471)	6 (1.3)	465 (98.7)	1			
Not circumcised (192)	9 (4.7)	183 (85.3)	3.8 [1.4 – 10.85]	0.001	3.79 [1.254-11.482]	0.01
***Alcohol***						
No (507)	5 (1.0)	502 (99.0)				
Yes (156)	10 (6.4)	146 (93.6)	6.87 [2.313 - 20.437]	0.001	6.33 [2.03-19.74]	0.001
***Number of partners***						
Single (141)	1 (0.71)	140 (99.3)	1			
Multiple (522)	14 (2.7)	508 (97.3)	3.85 [0.503 -29.5]	0.19	2.42 [0.287-20.466]	0.41

**Table 5:** **Factors associated with HIV seropositivity at delivery: Logistic regression analysis**

**Patient’s characteristic**	***HIV Status***		**Univariate**		**Multivariate**	
	**Positive n (%)**	**Negativen (%)**	**OR [95% CI]**	**p-value**	**OR [95% CI]**	**p-value**
*Age*						
≥20 (582)	140 (6.87)	73 (90.1)	1			
<20 (81)	8 (9.88)	568 (93.13)	1.49 [0.255 – 3.300]	0.515	1.61 [0.710-3.649]	0.255
*Education*						
Formal (627)	44 (7.02)	583 (92.98)	1			
Informal (36)	4 (11.1)	32 (88.9)	1.65 [0.600 - 4.894]	0.36	1.34 [0.413-4.194]	0.60
Syphilis results						
Negative (648)	42 (6.48)	606 (93.52)	1			
Positive (15)	6 (40.0)	9 (60.0)	9.61 [3.27 - 28.305]	0.0001	8.55 [2.718-26.198]	0.0001
*Partner circumcision*						
Circumcised (471)	32 (6.79)	439 (93.2)	1			
Not circumcised (192)	16 (8.33)	176 (91.6)	1.5 [0.667 – 2.330]	0.48	1.06 [0.554-2.054]	0.84
*Alcohol*						
No (507)	533 (6.5)	474 (93.4)				
Yes (156)	15 (9.6)	141 (90.4)	1.5 [0.806 - 2.893]	0.19	1.20 [0.606-2.415]	0.51
*Number of Partners*						
Single (141)	8 (5.67)	133 (94.3)	1			
Multiple (522)	140 (7.67)	482 (92.3)	1.37 [0.630 -3.018]	0.42	1.23 [0.556-2.748]	0.30

## Discussion

In this study, the prevalence of syphilis at delivery among pregnant women who did not test or tested negative for syphilis at ANC visits was 2.3%. This seroprevalence was lower than 9.3% reported in a study done in South Africa [21]. The low prevalence of syphilis observed in this study could be explained by good health care seeking behaviour of the study population as it was observed that 60% of women in this study attended ANC at least four times which is above the national average of 42%. When compared to the previous studies in the same region the prevalence is low. The decrease in syphilis seroprevalence has been observed in other studies done in Mwanza region, Kenya, Botswana and Ethiopia [22–25]. The syphilis seroprevalence of 1.8% among women who did not test at ANC was also lower than the 8% and 18.2% previously reported in Mwanza and South Africa [19,21] respectively. Overall there was no significant difference between seroprevalence of HIV at delivery among those tested during antenatal and those tested during delivery. This has been observed previously [26] in Nigeria.

In this study syphilis seroconversion rate was 2.7% while that of HIV was 2%. The findings are comparable to the study done in South Africa [21] with the same rate for syphilis and even lower levels have been reported elsewhere [27]. The HIV seroconversion rate observed in this study was inconsistent with a previous study [28] which reported seroconversion rate of 4.8%. This may be explained by well established HIV management and increased awareness among these women. The significance of retesting HIV late during the pregnancy is supported by previous studies [14,29,30] which indicated that some HIV positive babies are born to HIV seronegative mothers tested in early pregnancy.

In this study there were no association between syphilis seropositivity and maternal age during pregnancy (p = 0.515). Similar findings have been reported elsewhere in the country [31]. In addition, maternal level of education in this study was not associated with syphilis seropositivity in pregnancy (p = 0.353) which is inconsistent with previous study done in Tanzania where syphilis seroprevalence was associated with low level of education among pregnant women [32]. HIV seropositivity was significantly higher among syphilis seropositive pregnant women than among syphilis seronegative women (p < 0.001). Similar findings have been reported in other studies in northern Tanzania and in Kenya [33,34]. The syphilis and HIV co-infection rate of 0.9% observed in this study was lower than co-infection rates of 1.4% and 0.7% previously reported in other studies in Tanzania [24,32]. Male partner circumcision in this study was significantly associated with reduced syphilis seropositivity (p = 0.011), similar to what has been reported in study in Kenya [22]. In our study, syphilis seropositivity was significantly associated with history of alcohol consumption (p < 0.001), similar findings have also been reported in previous studies [35,36]. Alcohol intake may be one of the many health related risks such women undertake, including concurrent multiple sexual partnership and high rate of unprotected sex that predispose to acquisition of Sexually transmitted infections (STIs) including syphilis. Our study confirmed the association between ulcerative STIs and HIV as it was observed that women with syphilis were nine times more at risk of acquiring HIV infection than those without syphilis.

One of the major limitation of this study was recruiting only pregnant women attending ANC. This might influence the seroprevalence of syphilis and HIV due to the fact that women with risk behaviours might not wish to attend ANC because of the routine screening of these infections.

## Conclusion

Findings from this study suggest that women identified as negative for syphilis and HIV during ANC visits may seroconvert in the course of pregnancy and could benefit from re-screening. Delivery at health care facility also provides an opportunity for screening and treatment of women who missed out during the course of pregnancy. Seroconversion rates for syphilis and HIV observed in this study suggest that these women may potentially transmit these infections to the fetuses and affect the pregnancy outcome. This in turn justifies the need for re-screening for these infections especially in endemic areas in the third trimester and at delivery to reduce adverse perinatal outcomes associated with these infections.

## Abbreviations

ANC: Antenatal clinic
BMC: Bugando medical centre
CREC: CUHAS/BMC Research Committee
HIV: Human immunodeficiency virus
PMTCT: Prevention of mother to child transmission
RPR: Rapid plasma regain
STI’s: Sexually transmitted infections
WHO: World’s Health Organization
